# Comparison of a New P2Y12 Receptor Specific Platelet Aggregation Test with Other Laboratory Methods in Stroke Patients on Clopidogrel Monotherapy

**DOI:** 10.1371/journal.pone.0069417

**Published:** 2013-07-02

**Authors:** Zsuzsa Bagoly, Ferenc Sarkady, Tünde Magyar, János Kappelmayer, Endre Pongrácz, László Csiba, László Muszbek

**Affiliations:** 1 Clinical Research Center, University of Debrecen, Medical and Health Science Center, Debrecen, Hungary; 2 Department of Neurology, University of Debrecen, Medical and Health Science Center, Debrecen, Hungary; 3 Institute of Laboratory Medicine, University of Debrecen, Medical and Health Science Center, Debrecen, Hungary; 4 Szent Imre Hospital, Budapest, Hungary; 5 Thrombosis and Haemostasis Research Group of the Hungarian Academy of Sciences, Debrecen, Hungary; King’s College London School of Medicine, United Kingdom

## Abstract

**Background:**

Clinical studies suggest that 10-50% of patients are resistant to clopidogrel therapy. ADP induced platelet aggregation, a widely used test to monitor clopidogrel therapy, is affected by aspirin and is not specific for the P2Y12 receptor inhibited by clopidogrel.

**Objectives:**

To develop a P2Y12-specific platelet aggregation test and to compare it with other methods used for monitoring clopidogrel therapy.

**Patients/Methods:**

Study population included 111 patients with the history of ischemic stroke being on clopidogrel monotherapy and 140 controls. The effect of clopidogrel was tested by a newly developed ADP(PGE1) aggregation test in which prostaglandin E1 treated platelets are used. Results of conventional ADP induced platelet aggregation, VerifyNow P2Y12 assay and ADP(PGE1) aggregation were compared to those obtained by flow cytometric analysis of vasodilator stimulated phosphoprotein (VASP) phosphorylation. Reference intervals for all assays were determined according to the guidelines of Clinical Laboratory Standards Institute.

**Results:**

The P2Y12-specificity of ADP(PGE1) test was proven by comparing it with ADP aggregation in the presence of P2Y1 antagonist, adenosine 3’, 5’-diphosphate. The method was not influenced by aspirin treatment. Approximately 50% of patients were clopidogrel resistant by conventional ADP aggregation and VerifyNow tests. The ADP(PGE1) method and the VASP phosphorylation assay identified 25.9% and 11.7% of patients as non-responders, respectively. ADP(PGE1) aggregation showed good correlation with VASP phosphorylation and had high diagnostic efficiency.

**Conclusion:**

The new ADP(PGE1) method is a reliable test for monitoring P2Y12 receptor inhibition by platelet aggregation. As a subset of patients are non-responders, monitoring clopidogrel therapy by adequate methods is essential.

## Introduction

Clopidogrel, an irreversible inhibitor of platelet P2Y12 ADP receptor, is widely used as monotherapy or in combination with aspirin to reduce the risk of recurrent atherothrombotic ischemic events [1]. Clopidogrel is a pro-drug; its active metabolite is produced by the liver in a multistep process. The active metabolite covalently binds to the P2Y12 receptor and thereby inhibits the amplification mechanism of ADP-induced platelet activation and aggregation. Despite its potent antiplatelet effect, clinical studies suggest that approximately 10-50% of patients are resistant to therapy and it is not clear, which laboratory test is most suitable to identify such patients [2–5]. A number of methods are available for monitoring the effect of clopidogrel. For the time being, ADP-induced platelet aggregation, the most commonly used method, is considered as the gold standard [4,6,7]. One major drawback of this method is that it is not specific for P2Y12 receptor inhibition and aspirin therapy influences its effect. Despite this fact, most studies on clopidogrel resistance include patients on combined antiplatelet therapy (aspirin+clopidogrel) and only few reports are available on patients taking clopidogrel as monotherapy. Other methods, which are specific for P2Y12 receptor inhibition, such as the flow cytometric assay of vasodilator stimulated phosphoprotein (VASP) phophorylation and the VerifyNow P2Y12 tests are relatively expensive and require special instrumentation. A common problem with all of these methods is the lack of consensus cut-off values for identifying clopidogrel non-responders, which makes the interpretation of the data ambiguous [4].

In this study, we had three major aims: 1/ to develop and validate a P2Y12 receptor specific ADP aggregation test for the detection of clopidogrel’s effect, 2/ to determine reference intervals for different methods used to evaluate the effect of clopidogrel, 3/ to compare the results of these laboratory tests obtained in patients receiving clopidogrel monotherapy.

## Patients and Methods

### Patient and control population

Study population included 114 patients with the history of non-cardiogenic ischemic cerebrovascular disease being on 75 mg/day clopidogrel therapy for at least one month and 140 sex-matched healthy controls not taking any medication influencing platelet function. A priori exclusion criteria were: aspirin/non-steroid anti-inflammatory drug therapy, chronic liver disease, hemoglobin concentration <80 g L^-1^, platelet count >500×10^9^ L^-1^ or <150×10^9^ L^-1^, acute infectious disease/antibiotic treatment, qualitative defects of platelet function or other types of hemorrhagic diathesis, major surgical procedure or major ischemic event within one month of enrollment, admitted non-compliance. In the case of non-responders the possibility of non-compliance during the study was investigated by an oral interview. Whenever non-compliance was suspected, measurements were repeated after a two-week period of drug administration. Due to proven non-compliance three patients were excluded from the study. Baseline characteristics of patients and controls are shown in Table 1.

**Table 1:** Characteristics of patients and controls.

	Patients	Controls		
Number	111	140		
Male gender	53 (47.7%)	68 (48.5%)		p=0.89
Age (years)	61.6±10.4	43.3±18		p<0.001
BMI (kg m^-2^)	25.97±7.9	24.03±3.79		p<0.001
	Diabetes mellitus	22 (20%)		
History of hypertension	80 (72%)	10 (7.1%)		p<0.001
Dyslipidemia	73 (65.7%)	2 (1.4%)		p<0.001
Current smoker	24 (21.6%)	34 (24.2%)		p=0.64
	Previous MI	24 (21.6%)		
	History of multiple stroke/TIA	83 (74.7%)		
	PPI use	11 (9.9%)		
	Statin use	67 (60%)		
	Duration of clopidogrel therapy in months (median; range	12; 1-119		

BMI, body mass index; MI, myocardial infarction; PPI, proton pump inhibitor. Continuous data are presented as means ± standard deviation when normally distributed (age and BMI), statistical analysis was performed using Student’s t test. Duration of clopidogrel therapy showed non-parametric distribution and is expressed as median and range. Categorical variables are presented as counts (%); in this case differences between groups, where applicable, were tested by the χ^2^ test.

### Ethics statement

The study protocol was approved by the Scientific and Research Ethics Council of the Hungarian Ministry of Health (permission no. 8-281/2009-1018EKU). Written informed consent was obtained from all study participants.

### Blood sampling

Blood drawing was performed by venipuncture from an antecubital vein after overnight fasting. For light transmission aggregometry and VASP phosphorylation assay blood samples were collected into Vacutainer tubes containing 0.109 mol/L trisodium citrate (Becton-Dickinson, Franklin Lakes, NJ). For VerifyNow P2Y12 assay blood was collected into Vacuette tube (Greiner Bio-One, Basel, Switzerland). After blood drawing the VerifyNow P2Y12 assay, light transmission aggregometry and VASP phosphorylation test were performed within 1, 4 and 24 hours, respectively.

### Light transmission aggregometry and ATP release

ADP-induced platelet aggregation and secretion was monitored using a Chrono-Log model 700 lumiaggregometer (Chrono-Log Corporation, Havertown, PA). Citrate-anticoagulated whole blood was centrifuged at 150 g for 15 min at room temperature to obtain platelet rich plasma (PRP). After carefully removing the upper two third of PRP, tubes were further centrifuged at 1500 g for 20 min to obtain platelet poor plasma (PPP). Platelet count was adjusted by the addition of the required amount of PPP, to obtain a platelet count of 250×10^9^ L^-1^. Baseline optical density was set on PPP. Aggregation induced by 5 µM and 20 µM ADP (Helena Laboratories, Beaumont, TX) was monitored for 6 min. Luciferin-luciferase reagent (Biothema AB, Handen, Sweden) was added to each sample for the measurement of ATP release from platelet delta granules. Maximal percentage aggregation (maximal Δtransmission %) and ATP release (μmol ATP/10^11^ platelets) were recorded for each sample.

### P2Y12 specific ADP(PGE1) platelet aggregation test

Conventional ADP-induced platelet aggregation was modified in order to obtain P2Y12 receptor specific platelet aggregation. In this case PRP was pre-incubated with 0.31 µM prostaglandin E1 (PGE1; Sigma-Aldrich) for 3 min at 37 °C prior to ADP-induced aggregation to suppress the undesirable contribution of P2Y1 receptors. The extent of P2Y1 receptor suppression by PGE1 was compared to the effect of P2Y1 antagonist adenosine 3’, 5’-diphosphate (A3P5P; Sigma-Aldrich) [8]. In preliminary experiments, modified ADP-induced platelet aggregation was carried out using 20 µM, 40 µM and 60 µM ADP: optimal aggregation was observed with 40 µM or 60 µM ADP; 40 µM ADP was used throughout the study. The effect of aspirin treatment on the results was tested on 50 subjects being on 100 mg/day aspirin monotherapy.

### VerifyNow P2Y12 assay

The VerifyNow P2Y12 assay (Accumetrics, San Diego, CA) is a whole blood point-of-care test [9], which measures the ADP-induced co-agglutination of platelet and fibrinogen-coated beads in the presence of PGE1. The presence of PGE1 makes the test specific for the P2Y12 receptor pathway. In the assay ADP-activated platelets bind to fibrinogen-coated beads and agglutinate them. The instrument measures the change in light transmittance and reports results as P2Y12 reaction units (PRU). In addition, the device calculates the percentage of P2Y12 inhibition, based on thrombin receptor-activating peptide (TRAP)-induced platelet aggregation. However, in most publications the results are expressed as PRU.

### Flow cytometric analysis of vasodilator-stimulated phosphoprotein (VASP) phosphorylation

The VASP phosphorylation assay was carried out according to the manufacturer’s instructions using a commercially available kit from Biocytex (Marseille, France). The test is based on the fact that in the presence of PGE1 and ADP, the extent of VASP phosphorylation is proportional to the inhibition of platelets by clopidogrel [10]. Briefly, samples of citrate-anticoagulated blood were incubated with either PGE1 or PGE_1_+ADP and fixed in paraformaldehyde. Platelets were then permeabilized, labeled with a CD61 phycoerythrin-labeled platelet specific antibody and a FITC-labeled phosphorylated VASP (VASP-P) specific mouse monoclonal antibody or a negative isotype control antibody. Samples were analyzed on a FacsCalibur flow cytometer (Becton Dickinson, Franklin Lakes, NJ). Geometric mean fluorescence intensity (MFI) values were determined in the presence of PGE1 without or with the addition of ADP. The extent of VASP phosphorylation was expressed as platelet reactivity index (PRI) calculated from the MFI values (after deduction of MFI obtained with isotype control) using the following formula:

PRI(%)=100×[MFI_(PGE1)_ -MFI_(PGE1+ADP)_]/MFI_(PGE1)_

### Determination of reference intervals and statistical analysis

Statistical analysis was performed using GraphPad Prism Software (La Jolla, CA). For all methods the reference interval corresponding to the 99% central interval was determined according to the guidelines of Clinical Laboratory Standards Institute (CLSI) [11]. The lowest value of the reference interval was used as the diagnostic cut-off: those patients, who were on clopidogrel therapy and had results within the reference interval were considered non-responders to the therapy. Data obtained by different methods were correlated according to Spearman’s rank correlation method. Differences between patients and controls were analyzed using the Mann-Whitney test, p < 0.05 was considered as significant. In the case of categorical variables differences between groups were tested with the χ^2^ test. The results of different methods on clopidogrel-treated patients were compared to those obtained by the VASP assay by calculating coefficients of determination (*r* ^*2*^) and diagnostic efficiencies (DE). In the latter case the sum of results identical by the VASP and the other investigated assay, i.e., responders by both assays plus non-responders by both assays, was divided by the number of investigated patients. DE was expressed as percentage.

## Results

### P2Y12 specific ADP(PGE1) aggregation test

**Figure 1:**
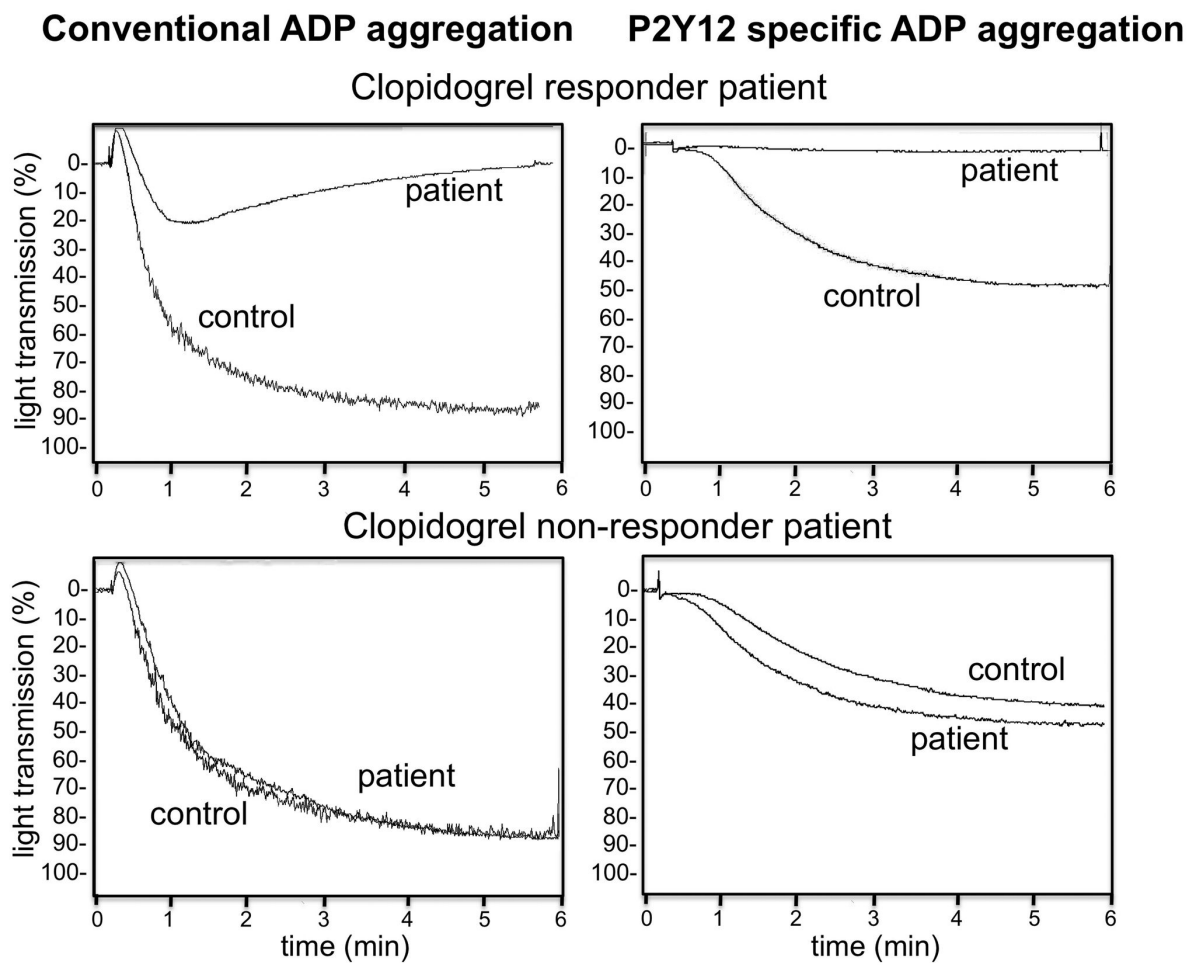
Representative ADP aggregation curves demonstrating the effect of clopidogrel treatment. Platelet aggregation was performed on PGE1 pretreated (right side) and non-pretreated (left side) platelet rich plasma (PRP). The upper and lower panels of the figure show results with PRP from a clopidogrel responder and a non-responder patient, respectively. Aggregation curves with samples from controls not taking antiplatelet medication are also demonstrated on each panel.

**Figure 2:**
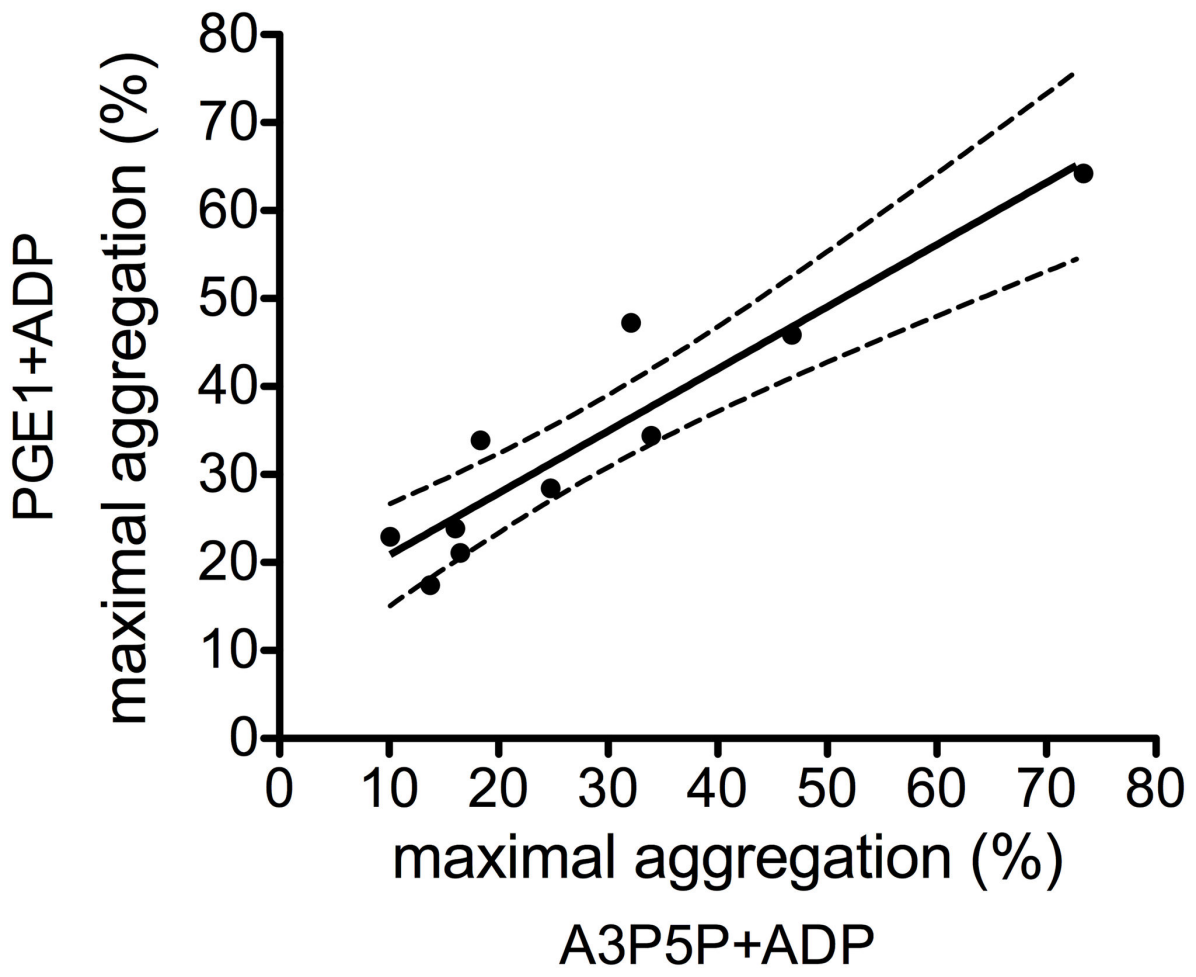
Correlation of ADP-induced aggregation in platelet rich plasma pre-treated with PGE1 or with P2Y1 inhibitor. Platelet rich plasma was pre-treated with 0.31 µM PGE1 or 1 mM adenosine 3’, 5’-diphosphate (A3P5P), a P2Y1 receptor inhibitor, for 3 min at 37 °C. Broken lines represent 95% confidence interval, r=0.89, p=0.001.

**Figure 3:**
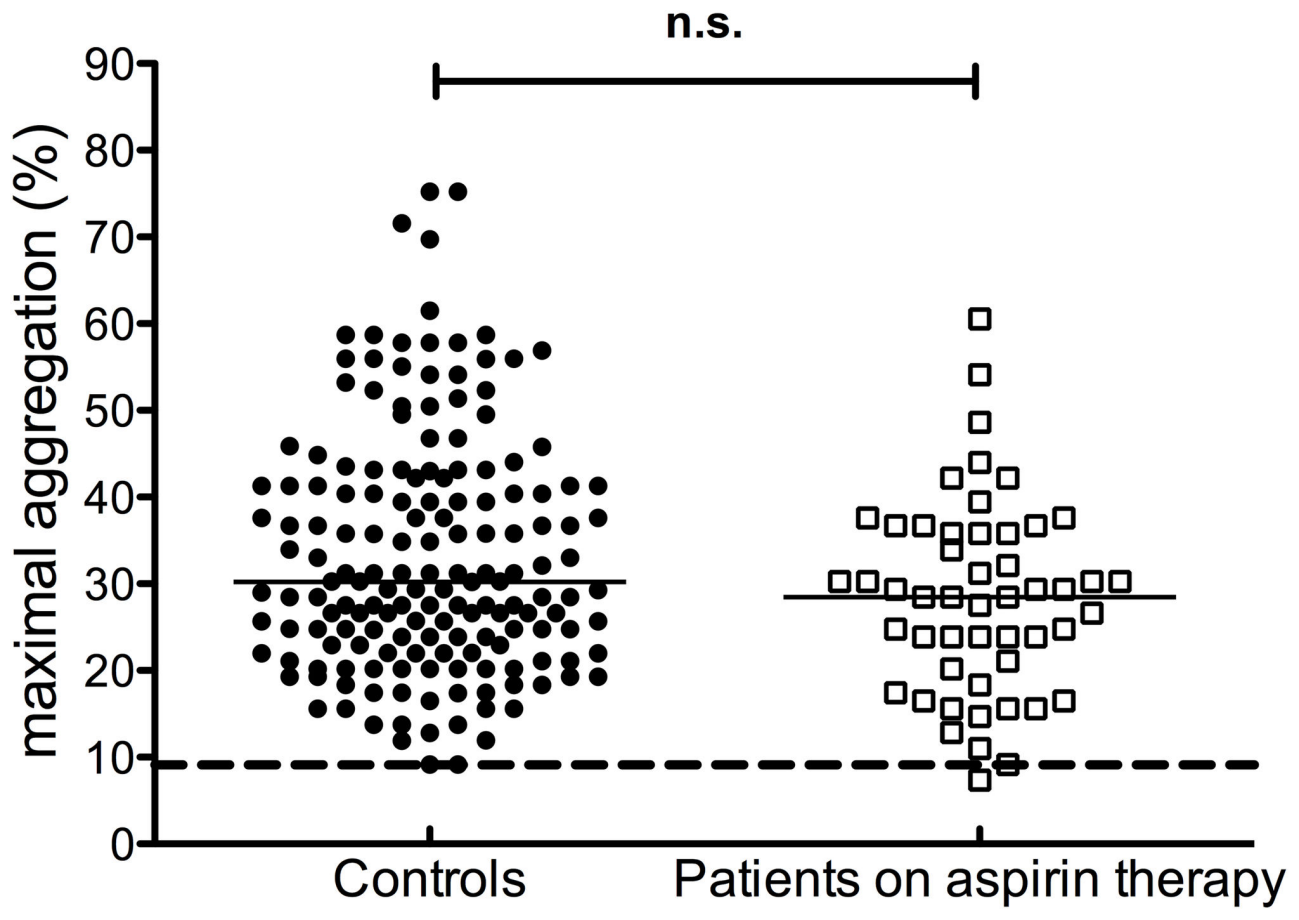
Aspirin therapy does not influence the P2Y12 specific ADP(PGE1) platelet aggregation test. ADP-induced aggregation in PGE1 pre-treated platelet rich plasma of controls (solid circles) and patients on aspirin therapy (open squares). Horizontal lines represent medians, the long broken line indicates the lower limit of reference interval. n.s.: non-significant.

Figure 1 demonstrates representative aggregation curves obtained with the ADP(PGE1) aggregation test and with the conventional ADP induced aggregation method. In case of a clopidogrel responder patient, residual transient aggregation was observed with the conventional ADP induced platelet aggregation method, due to the activation of P2Y1 receptor. When the PRP sample was pretreated with PGE1 the aggregation curve lacked the shape change signal and effective clopidogrel treatment completely abrogated ADP-induced aggregation. Aggregation of platelets from clopidogrel non-responder patients was comparable to that of control platelets. The effect of PGE1 pretreatment highly correlated (r=0.89, p=0.001) with that of the P2Y1 antagonist, A3P5P (Figure 2) demonstrating that the addition of PGE1 abolished signaling through the P2Y1 receptor. Another problem in testing the effect of clopidogrel by conventional ADP induced aggregation is its inhibition in patients on aspirin therapy. For this reason this test can hardly be used for testing the effect of clopidogrel on patients being on dual therapy. In contrast, the results of ADP(PGE1) aggregation obtained on PRP of controls and patients on aspirin monotherapy showed no difference (Figure 3).

#### Results of testing patients on clopidogrel monotherapy by different laboratory methods

**Figure 4:**
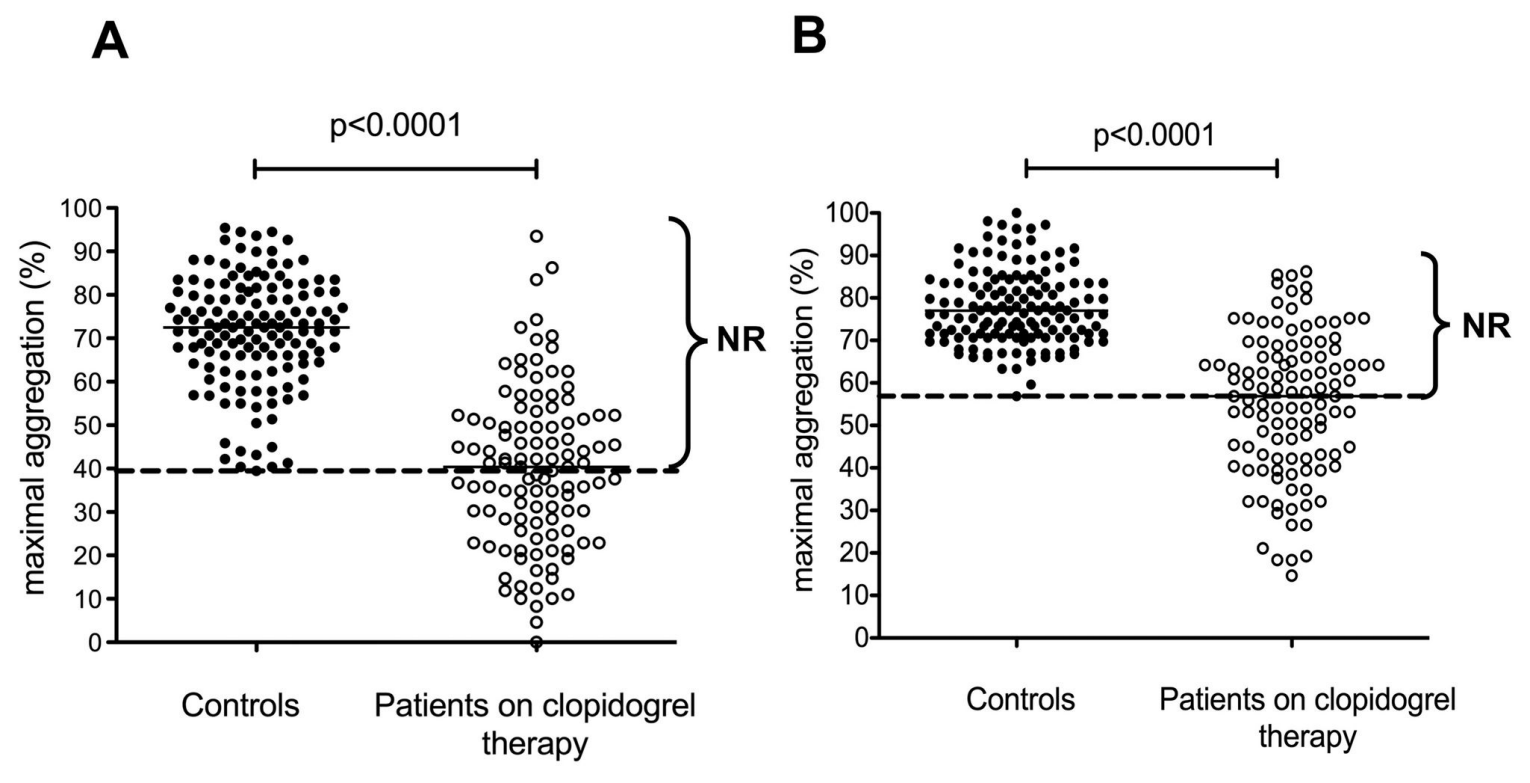
Results of ADP-induced platelet aggregation. Platelet aggregation was induced by 5 µM (A) and 20 µM ADP (B) in the platelet rich plasma of controls (solid circles) and patients on clopidogrel monotherapy (open circles). Horizontal lines represent medians, the long broken line indicates the lower limit of reference interval. NR: non-responder.

**Figure 5:**
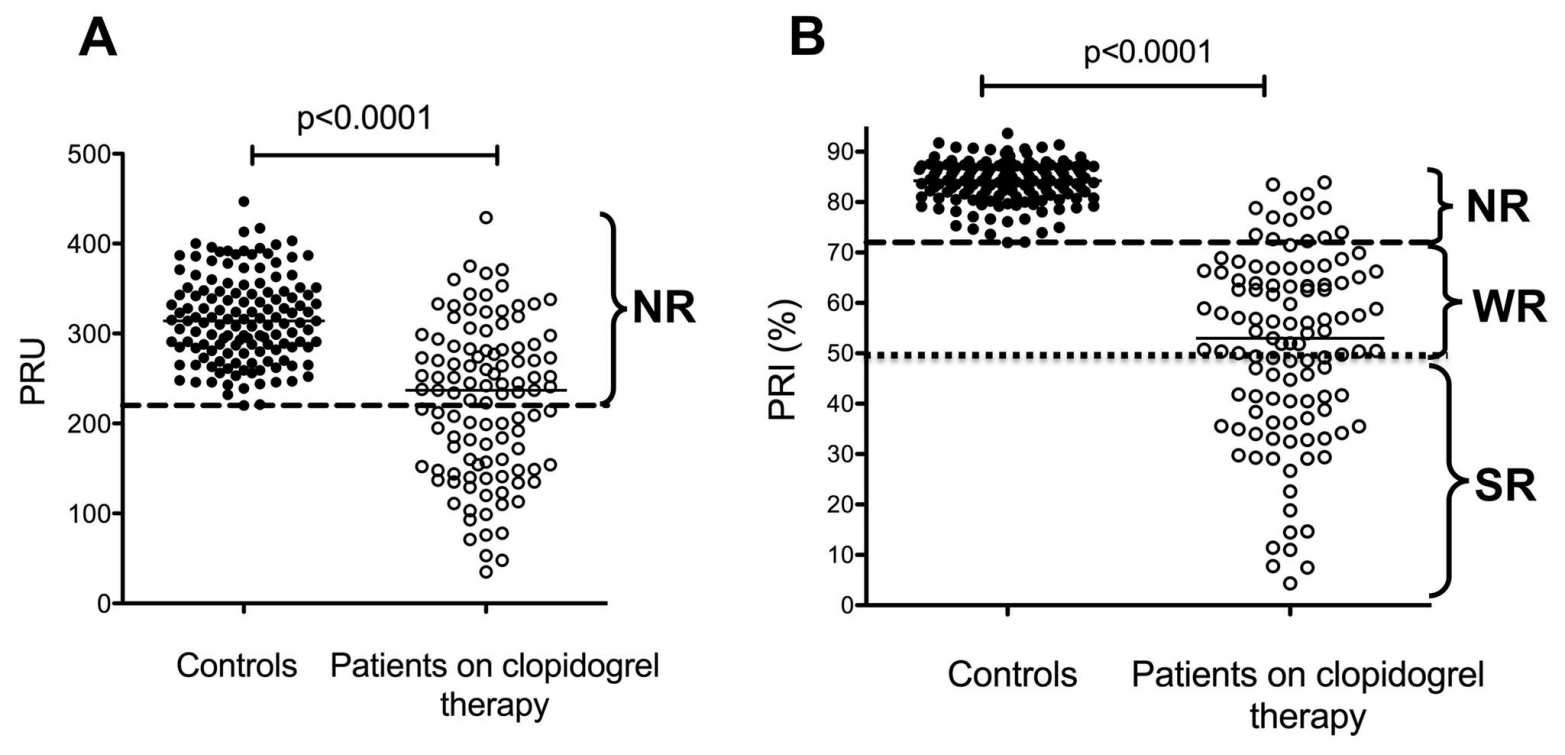
Results of VerifyNow P2Y12 and VASP phosphorylation tests. VerifyNow P2Y12 (A) and VASP phosphorylation (B) tests were performed in the control group (solid circles) and in the group of patients on clopidogrel therapy (open circles). Horizontal lines represent medians, the long broken line indicates the lower limit of reference interval, the long dotted line on panel B shows the cut-off for clopidogrel resistance established in clinical studies [4,5,12]. NR: non-responder, WR: weak responder, SR: strong responder.

**Figure 6:**
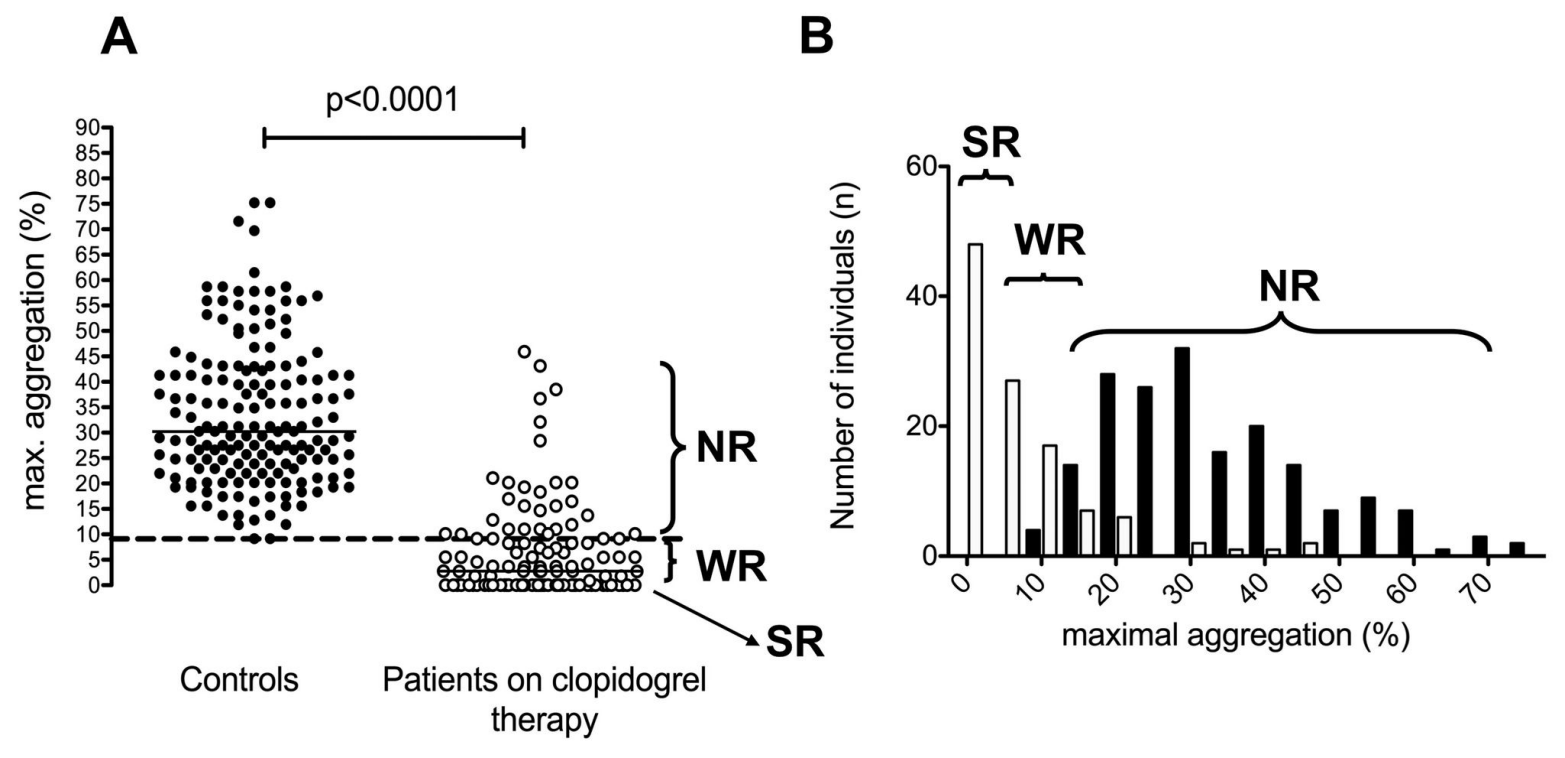
Results of P2Y12 specific ADP(PGE1) platelet aggregation test. P2Y12 specific ADP aggregation test was performed in the control group (solid circles) and in the group of patients on clopidogrel therapy (open circles). Results are demonstrated as scatterograms (A) and histograms (B). Horizontal lines on panel A represent medians, the long broken line indicates the lower limit of reference interval. NR: non-responder, WR: weak responder, SR: strong responder.

Cut-off values for clopidogrel non-responsiveness, corresponding to the lower limits of reference intervals, were: 39.5% maximal aggregation induced by 5 µM ADP, 56.8% maximal aggregation induced by 20 µM ADP, 220 PRU measured by the VerifyNow P2Y12 assay, 72% PRI as determined by the VASP phosphorylation test and 9.1% maximal aggregation induced by the ADP(PGE1) aggregation method (Figures 4-6). Based on these cut-off values the ratio of clopidogrel non-responders varied between 12–54% depending on the method used.

Approximately half of the patients were identified as non-responders by the traditional ADP aggregation method (50.5% using 5 µM and 51.4% using 20 µM ADP as agonist) (Figure 4). In the control group the results of ADP-induced ATP release scattered in a wide range (0-2.46 µmol ATP/10^11^ platelets with 5 µM ADP and 0.06-2.69 µmol ATP/10^11^ platelets with 20 µM ADP) including some very low secreted ATP values (data not shown). For this reason this method cannot be considered as the test for measuring the effect of clopidogrel.

The ratio of non-responders diagnosed by the P2Y12 specific VerifyNow P2Y12 assay (54%) was similar to that demonstrated by the conventional ADP aggregation method (Figure 5A). Using the other P2Y12 specific method, the VASP phosphorylation test, somewhat different results were obtained (Figure 5B). In this case the results of patients showed a wide distribution. Using the reference interval determined according to CLSI guidelines (72% PRI), 88.3% of the results of clopidogrel treated patients were below this limit indicating an effect specific to clopidogrel therapy. However, the wide distribution of results raised the possibility that some of these patients are not effectively protected by clopidogrel. In the past few years, a number of clinical studies involving patients on combined antiplatelet therapy suggested a cut-off of 50% PRI for clinical clopidogrel resistance [4,5,12]. Applying this cut-off to our patient population only 43.2% of patients would be in the clinically effective range. We considered these patients as strong responders, while patients with PRI below the reference interval, but above 50% were defined as weak responders.

The P2Y12 specific ADP(PGE1) platelet aggregation method was also tested on the study population (Fig. 6). Using this test, 28.8% of patients were identified as non-responders, i.e. their results were in the range of the control population. In 39.7% of patients no aggregation was observed, this group of patients was tentatively considered as strong responders, while 31.5% of the patients demonstrated impaired (< 9.1%), but detectable aggregation (weak responders).

### Correlation and diagnostic efficiency of laboratory tests used for the detection of clopidogrel effect

**Figure 7:**
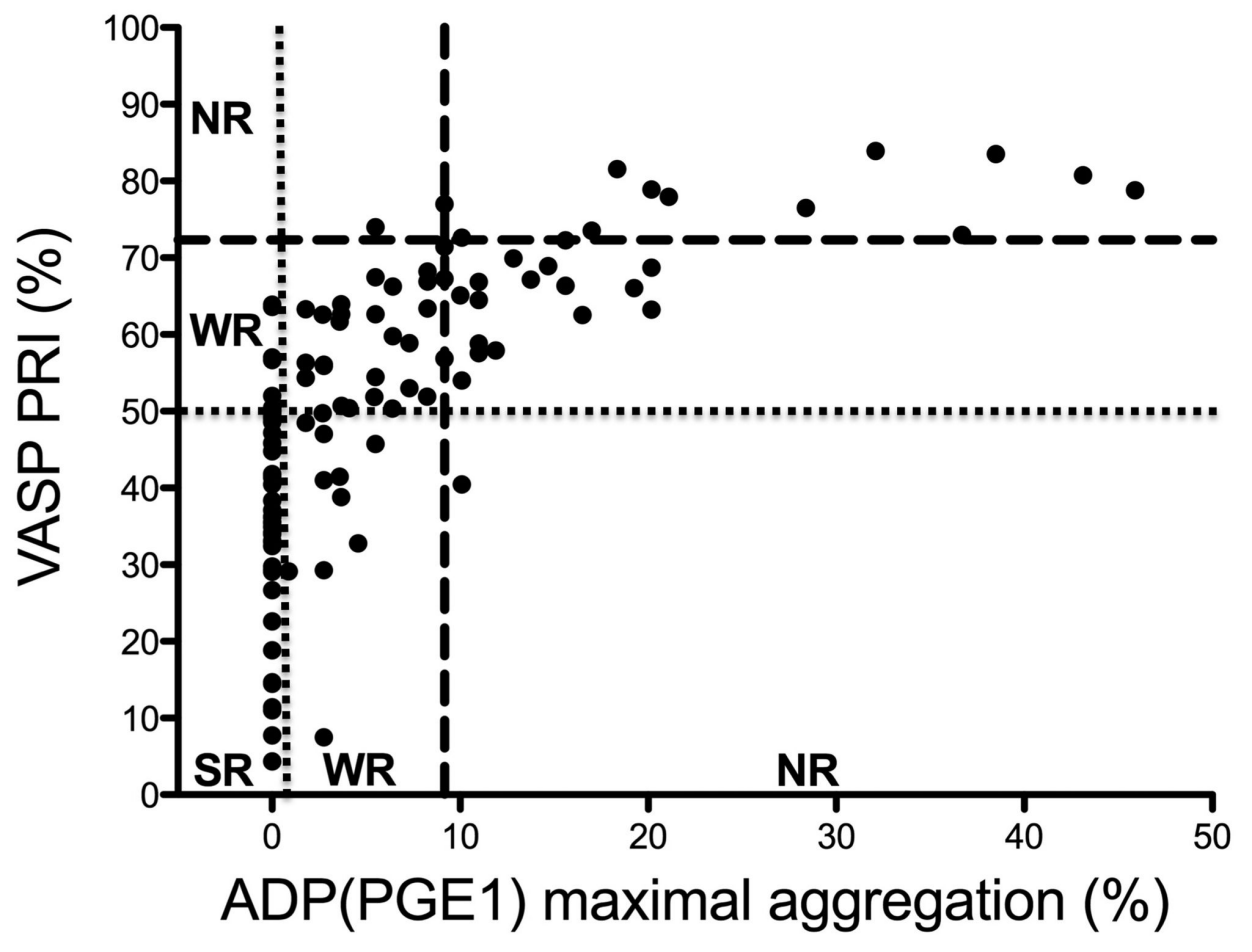
Correlation of VASP phosphorylation and P2Y12 specific ADP(PGE1) aggregation tests in clopidogrel treated patients. Broken lines represent the lower limit of reference intervals, dotted lines separate presumed strong responders from weak responders. NR: non-responder, WR: weak responder, SR: strong responder.

As the VASP assay is specific for P2Y12 inhibition and it was shown to have the best correlation with the plasma level of active metabolite [13,14], this assay was selected as the one to which other laboratory tests were compared. Among the investigated methods, the ADP(PGE1) aggregation assay had the highest DE (Table 2). The best correlation was found between the ADP(PGE1) aggregation test and VASP phosphorylation assay (r=0.79, r^2^=0.62, p<0.0001; Figure 7). The conventional ADP assays and the VerifyNow P2Y12 assay correlated with the VASP phosphorylation assay to a lesser extent (Table 2).

**Table 2:** Diagnostic efficiency and correlation of methods used for the measurement of platelet inhibition by clopidogrel.

	Diagnostic efficiency (%)	Coefficient of determination (r^2^)
5 µM ADP induced platelet aggregation	60.4	0.41
20 µM ADP induced platelet aggregation	60.4	0.43
VerifyNow P2Y12 test	56.9	0.50
ADP(PGE1) platelet aggregation method	85.6	0.62

Test results were compared to those obtained by the VASP phosphorylation assay. Results were calculated using the percentage of maximal aggregation and PRU in case of the platelet aggregation methods and the VerifyNow test, respectively.

## Discussion

Suboptimal inhibition of platelet function by clopidogrel in a considerable part of the patients has been demonstrated by a number of clinical studies [4,6,12,14–17]. To overcome the wide inter-individual variability in the response to clopidogrel, guided antiplatelet therapy has been suggested, which could improve clinical outcome and may result in a reduced rate of ischemic and hemorrhagic complications [12,18]. Tailored treatment based on the results of laboratory tests however is not yet adopted in routine clinical practice due to a number of important issues: questions of efficacy, cost-benefit ratios and lack of standardized laboratory test [14,16]. In order to monitor therapy, it is necessary to have an easily applicable, reliable, affordable method, which reports the specific effect of the antiplatelet drug. In addition, well established cut-off values are required for the adequate evaluation of laboratory results.

For the time being, ADP-induced platelet aggregation is still widely used to identify clopidogrel non-responders, although this test is not specific for the inhibition of the P2Y12 receptor and thus it is not optimal to test the effect of clopidogrel [4,7,14]. Moreover, most studies on clopidogrel resistance included patients on dual antiplatelet therapy (aspirin+clopidogrel) although aspirin inhibits ADP-induced platelet aggregation. This fact should always be taken into consideration when conventional ADP-induced platelet aggregation is used to evaluate the effect of clopidogrel in patients on dual antiplatelet therapy. It was demonstrated that the ADP(PGE1) method specifically detects the inhibition of P2Y12 receptor and avoids the undesirable contribution of P2Y1 receptor. It is to be noted that ADP aggregation, at agonist concentrations regularly used for aggregation (1-10 µM), is abolished by the blockade of P2Y1 receptor pathway. However, it was shown that much higher concentrations of ADP are capable of inducing platelet aggregation, but not shape change, in the PRP from P2Y1 knock out mice [19] and in human PRP in which P2Y1 receptor signaling was blocked by A3P5P [20]. Accordingly, we used 40 µM ADP in the newly developed test. The ADP(PGE1) method is not influenced by aspirin therapy, thus it is suitable to monitor clopidogrel responsiveness in patients on dual antiplatelet therapy. The test is inexpensive, relatively easy to carry out and does not require instrumentation other than an aggregometer.

Another major problem concerning the laboratory evaluation of clopidogrel therapy is the lack of consensus cut-off values. There could be two different definitions, laboratory and clinical, for the cut-off value. Non-responders defined by a laboratory method represent clopidogrel treated patients with laboratory results remaining in the reference interval established on healthy controls not taking antiplatelet medication. Values below the lower limit of reference interval prove an effect of clopidogrel, although this effect might vary widely to include weak to strong responses. In spite of numerous reports on clopidogrel resistance, well-established method-specific reference intervals for respective platelet function tests are scarcely available. In this study, we established reference intervals according to the guidelines of CLSI for all investigated methods. The use of reference interval based laboratory cut-off values allows the demonstration of the clopidogrel effect, but the demonstration of such effect does not necessarily mean effective protection against cerebrovascular events. Given the large inter-individual variability and the number of possible underlying mechanisms of variable platelet response to clopidogrel (variable absorption or metabolism, CYP polymorphisms, concomitant drug interaction, non-compliance etc. [21,22]) it might be important to distinguish patients who are not responding to therapy at all from patients showing moderate effects, although the clinical relevance of weak response to clopidogrel is yet to be determined. In several reports, based on the extent of response to clopidogrel as compared to pre-treatment values or to placebo controls patients were categorized as „non-responsive“, „semi-responsive“ and „responsive“ [23,24] or „high“, „average“ and „low“ responders [25,26]. However, such set-ups are rarely available in everyday clinical practice. Besides, the absolute level of on-treatment platelet reactivity seems to be a better measure of thrombotic risk than the change of platelet reactivity related to pretreatment values. Based on our results, categorization within the group of responders would be artificial, perhaps with the exception of the ADP(PGE1) method. In the latter case patients with complete inhibition of platelet aggregation were considered as strong responders, while patients showing aggregation below the reference interval were classified as weak responders.

In the past few years, a number of studies were published in which high on-treatment platelet reactivity was linked to ischemic events and clinical cut-offs were determined by receiver-operating characteristic (ROC) curves [4,12]. It should be noted that these cut-offs most likely depend on the subset of patients studied and to date, cut-off values have been estimated mainly in patients undergoing percutaneous coronary interventions (PCI). Interestingly, laboratory cut-offs established in our study were fairly similar to those obtained by a number of prospective studies in the case of ADP induced platelet aggregation and the VerifyNow P2Y12 assay [4,12]. For instance, in a large, prospective, observational study, including 1,069 consecutive patients treated with clopidogrel following elective PCI, cut-off points were comparable to those in our study: 42.9% vs. 39.5% for maximal aggregation induced by 5 µM ADP; 64.5% vs. 56.8% for maximal aggregation induced by 20 µM ADP; 236 PRU vs. 220 PRU for the VerifyNow P2Y12 assay [27]. In the case of VASP phosphorylation assay our study indicates that patients with results below the threshold of 72% PRI demonstrate a response to clopidogrel therapy, although it is possible that moderate response is unsatisfactory in a clinical setting. This cut-off is considerably higher than the most commonly used cut-off value based on the results of prospective studies (50% PRI) [5]. Obviously, in addition to clopidogrel non-responsiveness a number of other factors contribute to the clinical outcome.

Several studies have shown relatively poor agreement among different laboratory tests to identify clopidogrel non-responders [28,29]. Most of these studies include patients on dual antiplatelet therapy and only scarce reports involving relatively few patients are available on clopidogrel monotherapy [30,31]. In fact, it is less known how laboratory tests correlate in patient populations not affected by aspirin therapy. Clopidogrel as monotherapy is a first-line choice for anti-platelet therapy in ischemic stroke patients for secondary prevention of atherothrombotic events [32–34]. In our study, we investigated 111 ischemic stroke patients on clopidogrel monotherapy and compared the results of different laboratory tests. The best correlation was found between the ADP(PGE1) platelet aggregation test and the VASP phosphorylation assay. The highest DE of ADP(PGE1) platelet aggregation assay also indicates that this is a reliable test to monitor the efficacy of clopidogrel therapy.

Insufficient control of non-compliance, which mimics non-responsiveness, is a general limitation of such studies, including ours. Although considerable effort was made to detect non-compliance, it might not have been fully eliminated. This problem might influence the percent of non-responders, but does not influence method-to-method comparisons. Another limitation of our study could be the age difference between the control and patient group. We were not able to recruit sufficient number of age-matched controls not taking drugs potentially influencing platelet function. However, as test results did not show significant age dependence in the control group (not shown), age discrepancy is not likely to affect the results.

In conclusion, we have developed a new, reliable and affordable platelet aggregation method specific for the P2Y12 receptor and thus clopidogrel therapy. It showed good correlation with the VASP phosphorylation assay and had high DE in patients receiving clopidogrel monotherapy. As opposed to the conventional method, the new test is not influenced by aspirin therapy and therefore it is most likely a reliable choice to test patients on dual antiplatelet therapy. The value of this new test in a clinical setting remains to be investigated in prospective follow-up studies.
